# Impact of ZnO Addition on Er^3+^ Near-Infrared Emission, the Formation of Ag Nanoparticles, and the Crystallization of Sodium Fluorophosphate Glass

**DOI:** 10.3390/ma13030527

**Published:** 2020-01-22

**Authors:** Luukas Kuusela, Alexander Veber, Nadia G. Boetti, Laeticia Petit

**Affiliations:** 1 Photonics Laboratory, Tampere University, Korkeakoulunkatu 3, 33720 Tampere, Finland; luukas.kuusela@tuni.fi (L.K.); laeticia.petit@tuni.fi (L.P.); 2 Fondazione LINKS—Leading Innovation & Knowledge for Society, Via P. C. Boggio 61, 10138 Torino, Italy; nadia.boetti@linksfoundation.com

**Keywords:** glass, glass-ceramic, silver nanoparticles, erbium, luminescence, crystallization

## Abstract

The impact of the progressive addition of ZnO up to 5 mol% on the thermal, structural, and optical properties of Er^3+^-doped phosphate glasses within the system NaPO_3_-NaF-ZnO-Ag_2_O is discussed. The glass network was found to depolymerize upon the addition of ZnO. This promotes a slight increase in the intensity of the emission at 1.5 µm as well as enhances the silver ions clustering ability under the heat treating. The Ag-nanoparticles formed after moderate heat-treatment can further enhance the emission at 1.5 µm, whereas an excessive amount of the clusters leads to the opposite effect. The addition of ZnO helps to slightly increase the glass ability of the system. The crystallization behavior study revealed that surface crystallization is observed for all the glasses. It is found that even a small ZnO addition changes the crystalline phases formed after devitrification. Moreover, the addition of ZnO decreases the crystallization tendency of the glass.

## 1. Introduction

The addition of rare-earth elements (REE) into glasses with various compositions has been intensively studied, as these active glasses can find applications in telecommunications, light detection and ranging, solar panels, and bio-imaging, just to cite a few [1,2,3,4]. Of particular attention are the Er^3+^ due to the ^4^I_13/2_ →^4^I_15/2_ transition, which corresponds to an emission band at around 1530 nm. Er^3+^-doped phosphate glasses have been intensively investigated for many years, as phosphate glasses possess lower glass transition (*T_g_*), melting temperatures, and also good UV transparency compared to most of the silicate glasses [5]. Additionally, phosphate glasses have low dispersion, high possible rare-earth ions doping concentration, large refractive indices, and emission cross-section [6]. Therefore, Er^3+^-doped phosphate glasses are promising for amplifiers, wavelength division multiplexing, and optical communication systems operating at 1.5 μm [7,8,9].

Glasses are known to be a good medium for growing metallic nanoparticles (NPs) [10]. Different methods can be used to grow the metallic NPs such as sol–gel [11], direct metal-ion implantation [12], light-ion irradiation in ion-exchanged glasses [13], and annealing in hydrogen atmosphere of ion-exchanged glasses [14], just to cite a few. Most of these methods consist of two-steps: (i) the inclusion of metal ions into the glass matrix and (ii) the formation of clusters upon a post-treatment. Due to its simplicity and low cost, the thermal heat treatment method has been intensively used to form metal NPs in glass, especially when the glass is doped with rare-earth ions [15,16,17]. Rare-earth-doped glasses containing metallic NPs are attractive because the presence of NPs can enhance the luminescence [18] and the nonlinear properties [19] which are important for optical device applications. In particular, the formation of Ag nanoparticles can improve the spectroscopic properties of Er^3+^-doped glass due to the strong local electric field induced by surface plasmon resonance (SPR) of Ag NPs and also by energy transfer from the surface of silver NPs to Er^3+^ ions [20].

The other materials which can exhibit enhanced optical properties (such as absorption and emission cross-section and energy transfer rates) compared to glass are glass-ceramics (GCs). The enhancement of the spectroscopic properties can be achieved if the rare-earth ions are located around the crystalline phase [21,22]. Therefore, glass-ceramics have been intensively studied as they combine the merits of crystals and glasses [23,24,25]. As for the formation of metallic NPs, glass-ceramics are usually obtained by heat-treating a glass above its glass transition temperature. 

Very few studies about the fabrication of Er^3+^-doped glass-ceramics which also contain Ag NPs have been reported. The purpose of this study was to investigate the impact of the glass composition on various glass properties, especially the spectroscopic properties, on the formation of Ag nanoparticles to enhance the emission centered at 1.5 µm and finally on the glass crystallization tendency in order to prepare new glass-ceramics. 

In this work, new Er^3+^-doped phosphate glasses containing Ag_2_O were prepared and heat treated in order to precipitate Ag NPs and obtain glass-ceramics. We discuss first the impact of the glass composition on the structure and the spectroscopic properties of the glasses and then the impact of the glass structure on the Ag nanoparticles formation. Finally, we present results related to the preparation and characterization of glass-ceramics. 

## 2. Materials and Methods

Glasses with the composition (97 − x) × (0.9NaPO_3_-0.1NaF)-xZnO-2.5Ag_2_O-0.5Er_2_O_3_ with x = 0, 1.25, 2.5, and 5 were prepared using standard melting process in air. The raw materials were (NaPO_3_)_6_ (Alfa Aesar, Tewksbury, MA, USA, 99.99%), NaF (Sigma Aldrich, Saint Louis, MO, USA, ≥99.0%), ZnO (Sigma Aldrich, Saint Louis, MO, USA, ≥99.5%), Ag_2_SO_4_ (Sigma Aldrich, Saint Louis, MO, USA, 99.999%), and Er_2_O_3_ (MV Laboratories Inc, Frenchtown, NJ, USA, 99.5%). The 15 g batches were melted in quartz crucibles for 5 min at temperatures ranging between 800 °C and 875 °C, depending on the glass composition. After quenching, the glasses were annealed in a furnace at 200 °C for 6 h to release the stress from the quench and cooled down slowly by switching off the heating elements (cooling rate about 1 K/min). 

The Archimedes’ method was used to measure the densities of the glasses with an accuracy at ±0.02 g/cm^3^. Ethanol was used as the immersion liquid.

The thermal properties were measured using a STA 449 F1 instrument (NETZSCH, Selb, Germany). Powdered glasses were used for the experiment, and the heating rate was 10 °C/min. The *T_g_* was determined as the inflection point of the endotherm which is the first derivative of the differential thermal analysis (DTA) curve. *T_x_* and *T_p_* were taken as the onset and at the maximum point of the first exothermic peak, respectively. The accuracy of the measurement is ±3 °C. 

The Raman spectra were measured using an inVia™ Qontor^®^ confocal Raman microscope (Renishaw, Gloucestershire, UK) using a 405 nm laser. The measurements were done using polished glass samples. Additionally, the IR spectra were collected with a PerkinElmer Spectrum One FTIR in attenuated total reflectance (ATR) mode on powdered glasses. The resolution used was 2 cm^−1^ and the spectra were obtained from the accumulation of 8 scans.

The absorption spectra were measured using a UV-VIS-NIR spectrophotometer (UV-3600 Plus, Shimadzu, Kyoto, Japan) with a resolution of 0.2 nm. The absorption coefficients were used to estimate the absorption cross-section *σ_abs_* (*λ*) using Equation (1):

(1) $$\sigma_{Abs}\left(\lambda \right)=\frac{2.303}{NL}\log\left(\frac{I_{0}}{I}\right),$$

where log(*I*_0_/*I*) is the absorbance, *L* is the thickness of the sample (in cm), and *N* is the rare-earth ion concentration (ions/cm^3^). The Er^3+^ ions concentration was calculated from the measured densities of the glasses. 

The emission spectra of the glasses were measured at room temperature with a 0.5 nm interval using a Jobin Yvon iHR320 spectrometer (HORIBA Jobin Yvon, Edison, NJ, USA) with a Hamamatsu P4631-02 detector (Hamamatsu Photonics K.K., Hamamatsu, Japan) and a FEL1300 long-pass filter (Thorlabs Inc., Newton, NJ, USA). The excitation was done by a monochromatic 976 nm single-mode fiber pigtailed laser diode (CM962UF76P-10R, Oclaro Inc., San Jose, CA, USA). The fluorescence lifetime of the Er^3+^:^4^I_13/2_ →^4^I_15/2_ transition was obtained by exciting the samples with a fiber pigtailed laser diode operating at a wavelength of 976 nm, recording the signal using a digital oscilloscope (Tektronix TDS350, Tektronic Inc., Beaverton, OR, USA). The detector used for this measurement was an amplified InGaAs detector (PDA10CS-EC, Thorlabs Inc., Newton, NJ, USA). 

Due to the nonexponential decay law observed for all the samples, two types of decay law analysis were done. The average lifetime (*τ_avg_, s*) of the decay was calculated numerically using Equation (2):

(2) $$\tau_{avg}=\int \frac{t\cdot I\left(t\right)}{I\left(t\right)}dt.$$

It is the simplest way to characterize a nonexponential decay without any assumption. The average lifetime allows us to directly compare the results between different samples, but it is hardly describing a sophisticated function of the observed decay law if it differs from exponential.

To shed more light on the processes lying behind the nonexponential decay behavior, the luminescence decay curves were fitted using a stretched exponent function, which is described by Equation (3):

(3) $$I\left(t\right)=I_{0}e^{-{\left(t/\tau \right)}^{\beta }},$$

where *τ* (*s*) is effective time and *β* (*dimensionless*) is a constant ranging from 0 to 1. The stretched exponential behavior can originate from different microscopic mechanisms, in particular, for the glasses it can be an evidence of a disorder in the optical center environment [26,27]. In this case, the *β* parameter can be considered as a mere of this disorder: *β* = 1 corresponds to a single-exponent decay and no variation of the site, whereas a decrease in *β* indicates higher dispersion in the local environment of the optical center.

The Panalytical EMPYREAN multipurpose X-Ray Diffractometer (PANalytical, Almelo, The Netherlands) with a nickel-filtered copper K_α_ radiation was used to measure the XRD pattern of the glasses. The samples were crushed into fine powder and spread over a “zero-background holder” Si-plate. The spectra were obtained using the Bragg–Brentano geometry and by rotating the sample holder around the Phi-axis at a constant speed of 16 revolutions per minute.

A scanning electron microscope (Crossbeam 540, Carl Zeiss, Oberkochen, Germany) and an EDS detector (X-MaxN 80, Oxford Instruments, Abingdon-on-Thames, UK) were used to image and analyze the composition of the GCs obtained within the study. 

## 3. Results and Discussion

**Figure 1:**
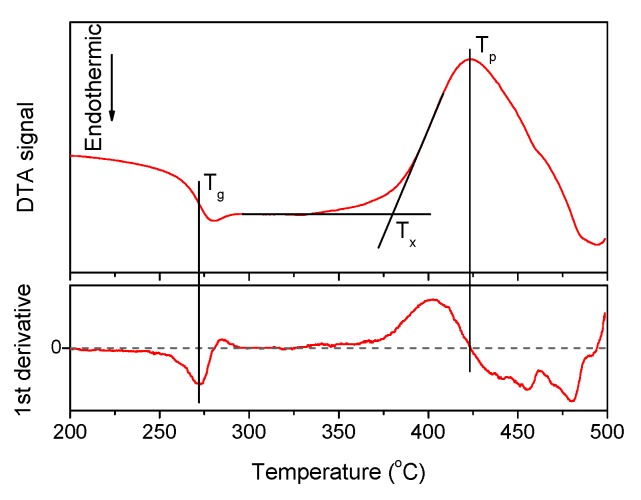
The DTA curve obtained for x = 1.25 glass, its first derivative. The characteristic temperatures of the material—*T_g_*, *T_x_*, and *T_p_*—are marked on the plot.

A typical DTA signal curve and the way to estimate the characteristic temperatures are shown in Figure 1, the densities and the thermal properties of the investigated glasses are given in Table 1.

An increase in Zn-content increases *T_x_*, *T_p_*, and the density due to the partial replacement of NaPO_3_ and NaF in the network by the heavier Zn. The change in the glass composition has no noticeable effect on *T_g_* but it increases the onset and the maximum of the crystallization temperature (*T_x_* and *T_p_*, respectively). Calculation of Δ*T = (T_x_ − T_g_)* values, which is a useful criterion for evaluating the thermal stability of the glass, reveals that all glasses exhibit Δ*T* larger than 100 °C, indicating that they are stable against crystallization and perspective for fiber drawing. Moreover, Δ*T* increases in Zn-containing glasses when compared to the Zn-free glass, evidencing that addition of Zn is favorable for enhancing the thermal stability of the glass.

**Figure 2:**
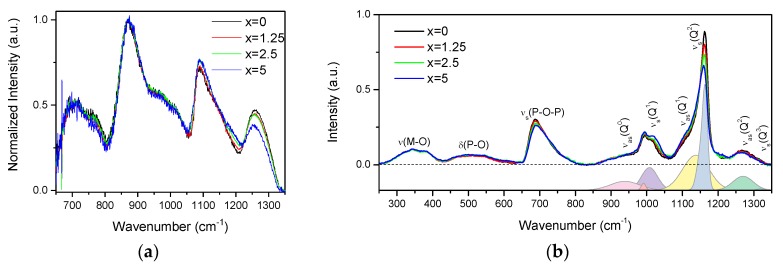
IR (**a**) and Raman (**b**) spectra of the investigated glasses. In the lower panel of the Raman spectra is deconvolution of the Q-species region obtained for x = 0 glass.

The IR and Raman spectra of the glasses are presented in Figure 2a,b, respectively, and are normalized to the main band centered at 870 cm^−1^ and to the area under the spectra, respectively.

The IR spectra (Figure 2a) exhibit bands at 650–800, 870, 1080, and 1260 cm^−1^, and various shoulders in the 730–800 and 930–1050 cm^−1^ ranges. The bands at 650–800, 830–1050, 1080, and 1260 cm^−1^ can be associated to *v_ss_(POP)*, *v_as_(POP)*, *v_ss_(POP)*, and *v_as_(OPO)* fundamental vibrations of *Q*^2^ units [28]. In the region of 830–1050 cm^−1^, the bands at 870, ~960, and ~1020 cm^−1^ are related to the asymmetric stretching vibrations of *Q*^2^ units in chains, small rings, and large rings, respectively [29,30]. The shoulder centered at ~980 cm^−1^ and the band at 1085 cm^−1^ correspond to the symmetric and asymmetric stretching vibration of *PO_3_^2−^* in *Q*^1^ units, respectively [29,30,31]. The *Q*^2^ and *Q*^1^ groups can also contribute to the bands at 1085 and 1190 cm^−1^, respectively [32]. An increase in x leads to the decrease in intensity of the band at 1260 cm^−1^ and to an increase in intensity of the band at 1085 cm^−1^, indicating an increase in the *Q*^1^ units at the expense of *Q*^2^ units. The decrease in intensity of the shoulder at 960 cm^−1^ and increase of the band at 870 cm^−1^ indicate that replacement of small phosphate rings by long chain structure of the glass occurs when x increases. 

The Raman spectra (Figure 2b) also present a superposition of the vibrational modes present in the glasses: the bands in the high-frequency region (850–1350 cm^−1^) originate from stretching modes of different Q-species present in the glass structure, namely, asymmetric PO_4_ stretch of *Q*^0^ tetrahedrons at 950 cm^−1^ (*ν_as_(Q^0^)*) [33]; symmetrical and asymmetrical stretching modes of *Q*^1^ at 1000 (*ν_s_(Q^1^)*) and 1140 cm^−1^ (*ν_as_(Q^1^)*) [33,34] and of *Q*^2^ units at 1165 (*ν_s_(Q^2^))* and 1270 cm^−1^ (*ν_as_(Q^2^)*) [35], respectively; and symmetrical stretch due to *Q^3^* species (*ν_s_(Q^3^)*/1320 cm^−1^) [33]. The mid-frequency band *ν_s_(POP)* (650–820 cm^−1^) is due to the symmetric stretching modes of *P–O–P* bridges (*ν_s_(P-O-P)*), and the lower frequency bands *δ(P-O)* (450–600 cm^−1^) and *ν(M–O)* (300–400 cm^−1^) are associated with bending modes of different structural units and the localized vibrations of metal cations in their oxide sites, respectively [35]. 

Relative contributions of the Raman bands in the glasses can be understood from the deconvolution of the Q-species frequency region into Gaussian components done for x = 0 glass (Figure 2b). The change in the glass composition mostly affects the Q-species and *P-O-P* bridges regions of the Raman spectra. Increase in Zn-content led to redistribution in the *Q^n^*-units: contributions of *Q*^1^ and *Q*^0^ units increase in intensity compared to the bands related to *Q*^2^ units. 

The observed changes in Raman and IR spectra with ZnO addition are consistent with each other, evidencing that Zn acts as a modifier and depolymerizes the phosphate network, leading to the formation of a less cross-linked network with a higher number of nonbridging oxygens (NBOs). 

**Figure 3:**
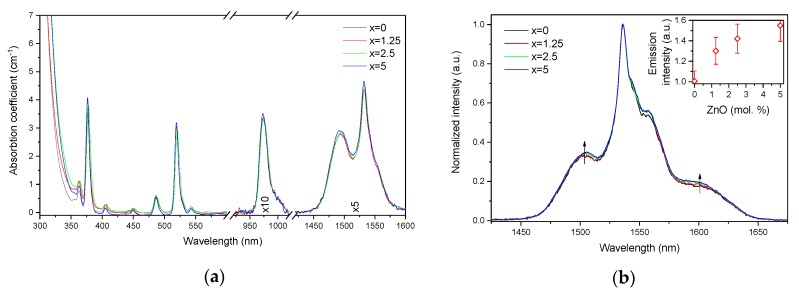
Absorption (**a**) and normalized emission (**b**) spectra of the investigated glasses. Integral emission intensity is shown in the inset. The emission spectra were obtained using *λ_exc_* = 976 nm.

The absorption and emission spectra of the investigated glasses are shown in Figure 3a,b, respectively. The absorption spectra exhibit the typical bands related to the transitions from the ground state ^4^I_15/2_ to different excited states of Er^3+^ ions. One should point out the absence of the absorption band at 400 nm, known as the SPR absorption band of Ag NPs [36,37], indicating that there are mostly Ag cations dispersed in the glass network and/or the amount of Ag NPs is not enough to give a noticeable signal. There is a slight shift of the UV absorption edge towards higher wavelengths as x increases. As mentioned above, at the ZnO concentrations used in this study, Zn acts as a network modifier, i.e., it causes progressive depolymerization of the network with the addition and increases the number of NBOs, which is known to reduce the bandgap energy [38]. At the same time, elements with d^10^ electronic configuration (Zn^2+^, Cu^+^, Ag^+^, etc.) show an intense absorption in short UV [39], and contribution from d-orbital electrons to the valence band can often explain the shift of the bandgap in the case of crystalline materials [40,41]. Therefore, the scenario that Zn ions may cause the valence band tailing and hence reduce the bandgap energy cannot be completely excluded here.

The absorption coefficients of the peaks at 975 nm and 1.5 µm were used to determine the absorption cross-sections using Equation (1). As shown in Table 1, the variation of the absorption coefficients and cross-sections at 975 nm and 1.5 µm between the samples does not exceed the experimental error of the measurement.

**Figure 4:**
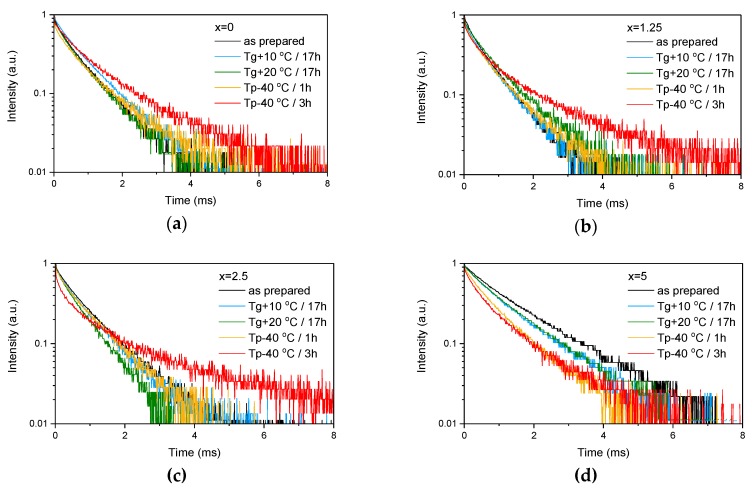
Emission decay curves of Er^3+^:^4^I_13/2_→^4^I_15/2_ optical transition measured for as-prepared as well as heat treated samples with various ZnO content: x = 0 (**a**), x = 1.25 (**b**), x = 2.5 (**c**) and x = 5 (**d**).

The emission spectra of the glasses, presented in Figure 3b, exhibit the typical luminescence of Er^3+^ ions in glasses, which corresponds to the transition from the ^4^I_13/2_ level to the ground state ^4^I_15/2_. An increase in x enhances the intensity of the emission at 1.5 µm. A similar impact of ZnO addition was reported in [42]. Previously, it was demonstrated that changes in the phosphate network under introduction of a network modifier affect the probability of REE-clusters formation in phosphate glasses [43]. Based on this, we assume that in case of the glasses under investigation, as the Zn depolymerizes the phosphate network, it is possible that the Er-Er distance becomes longer, slightly increasing the intensity of the emission. Besides the increase of the luminescence intensity, a slight change in the shape of the emission band as well as an increase of the average lifetime values of the Er^3+^:^4^I_13/2_ level (see Figure 4 and *τ_avg_*, Table 2) are observed. 

These provide evidence that changes in the glass structure due to Zn introduction also have some (minor) impact on the local environment of Er^3+^ ions. Previously, it has been shown that regularity of the site of an REE increases as polymerization decreases [44] and can be understood as “decoupling” of the Er-O group from the rest of the glass network [45]. Our experimental data show that the emission decay law becomes closer to exponential with the increase in ZnO content, which can be seen by the increase of the β-parameter (see Table 3, result of the fit is shown in Figure S1) obtained by the fit of the emission decay curves using Equation (3). This indirectly confirms increasing regularity of the Er^3+^ site with depolymerization of the glass network due to the Zn addition.

It is important to note that the lifetimes of the investigated glasses are shorter compared to those reported in fluorophosphate glasses (3 ms) [46], tellurite glasses (3.3 ms) [47], and also borosilicate glasses (2 ms) [48]. This is probably due to the large amount of OH^−^ groups expected in the glasses due to their high concentration in Na^+^ [49].

**Figure 5:**
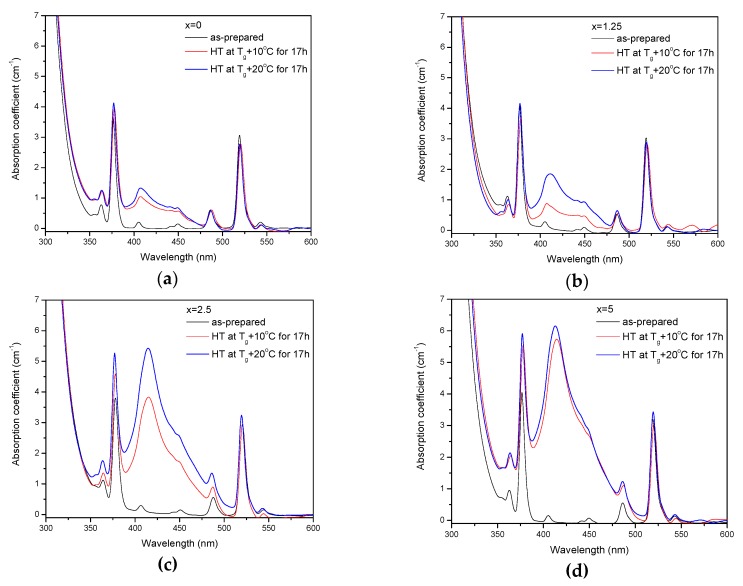
Absorption spectra of the glasses prior to and after heat treatment at *T_g_* + 10 °C and 20 °C for 17 h for x = 0 (**a**), x = 1.25 (**b**), x = 2.5 (**c**), and x = 5 (**d**).

The as-prepared glasses were heat treated at their respective *T_g_* + 10 °C and *T_g_* + 20 °C for 17 h. After the heat treatment, the color of the glasses changed from pink to yellow. The absorption spectra of the heat-treated glasses are shown in Figure 5. Compared to the spectra of as-prepared glasses, the spectra of the heat-treated glasses exhibit a new, broad band with maximum at ~410 nm, which overlaps with the Er^3+^ absorption bands and determines the final color. As explained earlier, this band corresponds to the surface plasmon resonance (SPR) absorption of Ag NPs [36,37]. 

**Figure 6:**
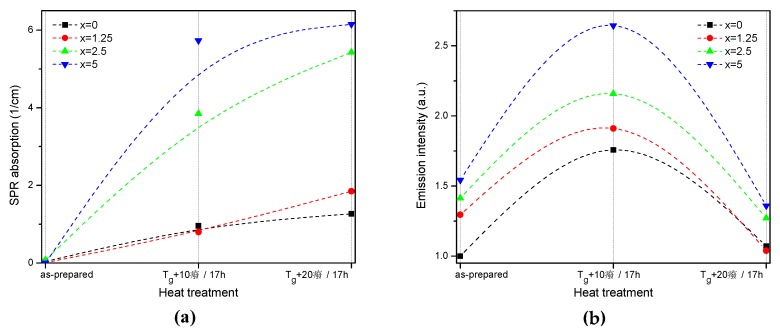
Absorption coefficient of the SPR band (**a**) and integral emission intensity of Er^3+^:^4^I_13/2_→^4^I_15/_2 optical transition (**b**) prior to and after the heat treatment at *T_g_* + 10 °C and 20 °C for 17 h of the glasses with x = 0, 1.25, 2.5, and 5. The experimental values are marked with symbols whereas the dashed lines serve as guides to the eye only.

The value of the absorption coefficient of this band at its maximum for different glasses is shown in Figure 6a. The intensity of this new absorption band increases as the temperature of the heat treatment increases. Intensity and position of the band depends on the size and concentration of the silver nanoparticles [50,51]. According to a previous study on a similar glass composition, in which size of the Ag NPs was determined directly using a transmission electron microscope [52], the observed position of the SPR absorption band should correspond to the Ag NP size of 20–40 nm range. The increase in Zn content also increases the intensity of this absorption band, indicating that Ag NPs form more easily in a more depolymerized phosphate network. The increase in the yield of the Ag NPs formation can be attributed to the increased mobility of Ag^+^ through the depolymerized glass lattice and also to the increase in the amount of NBO induced by the addition of ZnO in the phosphate network [53].

Although the heat treatment has no impact on the absorption coefficients and cross-sections of Er^3+^, the intensity of the 1.5 µm emission band increases after the heat treatment for 17 h at *T_g_* + 10 °C (see Figure 6b). The largest increase in emission induced by the heat treatment was observed for x = 5 glass, indicating that this glass is the most promising for practical applications. At the same time, some minor changes in the emission spectra of the heat-treated glasses are most pronounced for x = 0 and x = 1.25 glasses after the treatment at *T_g_* + 10 °C, whereas shapes of the emission band for all the other glasses reproduce that of the as-prepared glasses well (see Figure S2). Considering the fact that the excitation wavelength is far from the NPs absorption band, a direct energy transfer from the metallic NPs to RE ions is hardly possible. There are three mechanisms that could explain the enhancement of the Er^3+^ emission in the presence of metallic NPs: increase of the local incident field close to the optical center; increase of intrinsic radiative decay rate; or fluorophore-metal resonance energy transfer [54]. The excitation and emission wavelengths are far from the Ag NPs SPR band; therefore, the first mechanism should not be dominant. The latter two mechanisms should affect (decrease) the emission lifetime, which is also not observed in this study. Besides these three common mechanisms, it was shown that Me NPs can act as chemical absorbents of hydroxyl groups and increase the number of OH-free Er^3+^ ions, which should result in recovery of the emission lifetime [55]. The changes observed for average lifetime values (Table 2) depend on the Zn-content in the glass: a small increase is observed for x = 0, no variation found for x = 1.25, and some decrease was detected for x = 2.5 and 5 samples. This makes it difficult to identify the dominant mechanism responsible for the observed luminescence enhancement, which may also differ for glasses with different Zn concentration. 

Despite the fact that the heat treatment at higher temperature (*T_g_* + 20 °C) slightly increases the intensity of the SPR absorption band, this leads to a slight decrease in emission compared to the as-prepared glasses. Previously, it has been shown that Er^3+^-Ag NP distance plays a crucial role on the RE-ion luminescence intensity and excessive amount of the NPs in the optical center vicinity can serve as an additional nonradiative relaxation pathway [56]. The observed decrease in emission intensity could be due to the energy transfer from the exited states of Er^3+^ to the silver NPs, as suggested in [56].

The glasses heat treated at their respective *T_g_* + 20 °C for 17 h were also held at their respective *T_p_* − 40 °C for 15 min, 1 h, and 3 h. After this heat treatment, not only did the color of the glasses change from pink to orange, but the glasses also became opaque, which is a clear sign of crystallization. All glasses exhibit a ceramic appearance at their surface, indicating that surface crystallization occurs in all glasses, independently of their composition, upon heat treatment. The glasses with x = 2.5 and 5 were still translucent after 15 min at (*T_p_* − 40 °C) while the glasses with x = 0 and 1.25 were already opaque, indicating again that the addition of ZnO reduces the crystallization tendency of the glass. Similar impact of ZnO on the crystallization tendency of phosphate glasses was reported before in [57]. 

**Figure 7:**
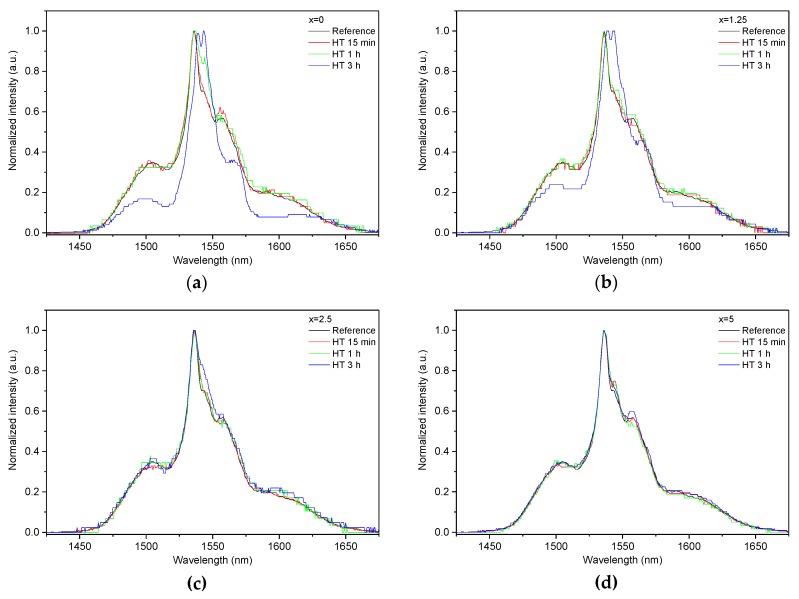
Normalized emission spectra measured at the surface of the glasses with x = 0 (**a**), x = 1.25 (**b**), x = 2.5 (**c**), and x = 5 (**d**) prior to and after heat treatment at *T_g_* + 20 °C for 17 h followed by a hold at their respective *T_p_* − 40 °C for 15 min, 1 h, and 3 h. The emission spectra were obtained using *λ_exc_* = 976 nm.

**Figure 8:**
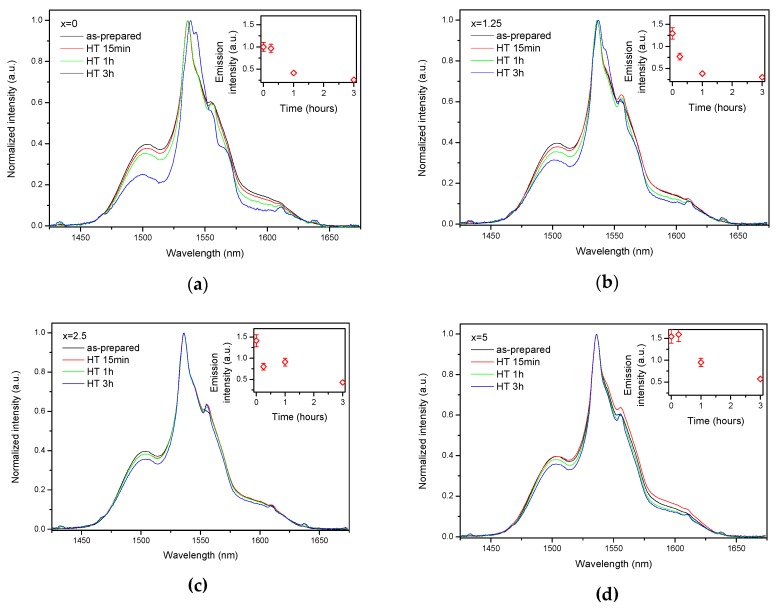
Normalized emission spectra of the glasses crushed into powder with x = 0 (**a**), x = 1.25 (**b**), x = 2.5 (**c**), and x = 5 (**d**) prior to and after heat treatment at *T_g_* + 20 °C for 17 h followed by a hold at their respective *T_p_* − 40 °C for 15 min, 1 h, and 3 h. Integrated emission intensities are shown in the insets. The error bars for the integral emission intensity data are not shown if the error does not exceed the size of the symbol. The emission spectra were obtained using *λ_exc_* = 976 nm.

As the glasses exhibit surface crystallization, at first the emission spectra were collected from the surface of the heat-treated glasses. As shown in Figure 7, the shape of the emission band of the glasses with x = 0 and 1.25 changes significantly, indicating that the site of the Er^3+^ ions in these glasses is modified after the heat treatment, whereas not so strong modification of the site is observed in the heat treated glasses with x = 2.5 and 5. To be able to compare the emission intensity between the samples, the luminescence spectra were also measured from the glasses crushed into powder prior and after the heat treatment. As seen in Figure 8, the intensity of the emission decreases after heat treatment for all the glasses, the strongest decrease was observed for the Zn-free glass. The differences in the emission spectra shapes measured from the surface and powder (see Figure 7, Figure 8, and Figure S3) once again confirm the surface crystallization mechanism of the glasses. In this case, the powdered samples present a mixture of crystalline and residual amorphous phases, which makes the changes in the shape of the emission spectra not so prominent when compared to the emission spectra measured from the highly crystalized surface layer. Significant changes were observed in the luminescence decay laws after the crystallization (see Figure 4). As seen from Table 3, the *β*-parameter decreases for all the compositions after the devitrification, in particular, after the 3 h treatment, indicating that the decay curves become highly nonexponential. This fact can be explained by redistribution of Er^3+^ among different sites of the obtained glass-ceramics.

**Figure 9:**
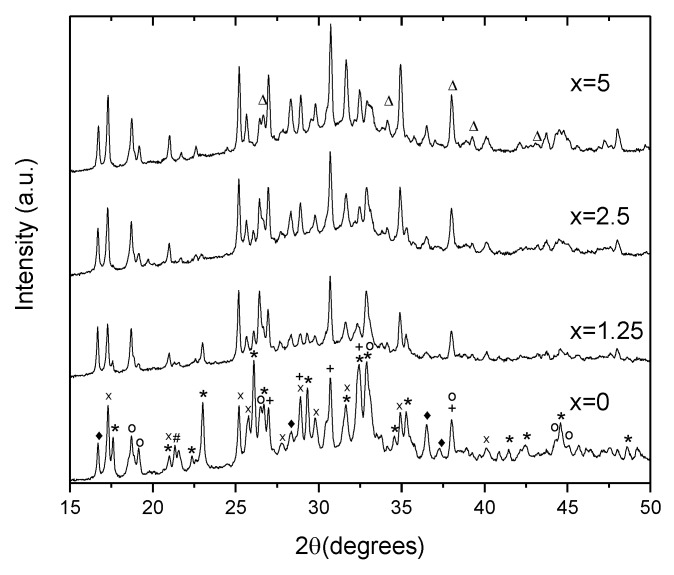
XRD pattern of the glasses heat treated at *T_g_* + 20 °C for 17 h followed by a hold at their respective *T_p_* − 40 °C for 3 h (*—NaPO_3_, **x**—Ag(PO_3_), **+**—Ag_4_P_2_O_7_, **o**—Na_5_P_4_O_10_, ♦—NaAgF_4_, **#**—NaAgO, and Δ—ZnF_2_).

To identify the crystals, XRD patterns of the glasses heat treated at *T_g_* + 20 °C for 17 h and (*T_p_* − 40 °C) for 3 h were measured from the crushed into powder samples. As shown in Figure 9, the XRD patterns obtained evidence that more than one crystal phase precipitate in the glasses during the heat treatment. Most of the peaks in the XRD pattern of x = 0 glass can be related to NaPO_3_ (JCPDS Card No. 04-011-5990), Ag(PO_3_) (JCPDS Card No. 98-026-1322), Na_5_(P_3_O_10_) (JCPDS Card No. 00-011-0652), NaAgO (JCPDS Card No. 04-010-7061), and NaAgF_4_ (04-013-8787). An increase in Zn decreases the amount of NaPO_3_ phase, whereas the peaks related to NaAgF_4_ and AgPO_3_ increase in intensity. After devitrification of the x = 5 glass, contribution of the NaPO_3_ phase can hardly be detected. Due to some additional minor changes observed in the XRD pattern, some zinc-bearing fluorine phases are also suspected here in Zn-containing glasses, in particular, ZnF_2_ and AgZnF_3_. Though the XRD patterns obtained do not allow us to precisely evaluate the amount of the amorphous and crystalline phases in the obtained GCs, the relative contribution of the crystalline part, calculated as a ratio of integrated area under the sharp peaks to the area under the whole pattern, decreases about 2 times, when comparing x = 0 and x = 5 glasses, respectively. This fact once again supports the conclusion that addition of ZnO reduces the crystallization tendency of the glass.

**Figure 10:**
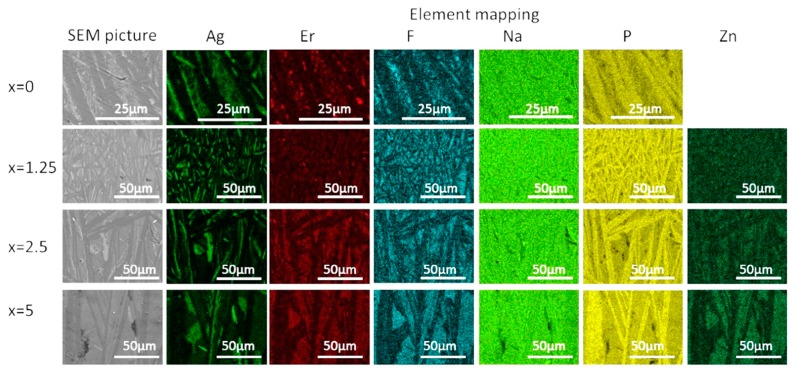
SEM image and elemental mapping of the crystalline layer in the glasses heat treated at *T_g_* + 20 °C for 17 h followed by a hold at their respective *T_p_* − 40 °C for 3 h.

The crystallized layer was additionally studied using SEM. As shown in Figure 10, crystals are clearly visible, their shape and size depend on the mother glass composition. The elemental mapping shows a redistribution of the elements between different phases obtained. One may see that Ag-, F-, and P-rich crystals precipitated during the heat treatment, whereas Zn-rich crystals are present in the heat-treated Zn-containing glasses. This confirms phase identification based on the XRD patterns analysis. It is important to note that fluorine content seems to be correlated with Ag in x = 0 glass—however, this changes upon the Zn addition, and F and Zn are clearly correlated in x = 2.5 and 5 glasses, indirectly confirming formation of Zn-bearing fluorine phases. The elemental mapping of Er tends to indicate that the rare-earth ions are redistributed between different crystalline phases, however, somewhat higher Er content is observed for the areas with higher fluorine content. 

The incorporation of Er^3+^ into a crystal lattice should significantly change the local environment of the active ions and influence their optical properties, as seen from the emission spectra of the devitrified samples which exhibit sharp peaks. However, relative contribution of these spectral features decreases with an increase of ZnO content. This can be explained by the fact that the addition of ZnO reduces the tendency of the glass to crystallize. From the XRD data, one can see that NaPO_3_ precipitates as the individual phase in glasses with low Zn-amount, probably leading to higher crystallization degree, especially in the case of x = 0 and 1.25 samples. The lack of the residual amorphous phase forces Er^3+^ to build into new crystalline phases. Higher ZnO amount leads to formation of Zn-bearing crystals, and most of the Er^3+^ ions are suspected to remain in the parent amorphous phase even after the heat treatment. This assumption could explain the increasing similarity of the emission spectra and lifetimes before and after the heat treatment from the glasses with increasing Zn-amount.

## 4. Conclusions

In this study, phosphate glasses were prepared with the composition (97 − x) × (0.9NaPO_3_-0.1NaF)-xZnO-2.5Ag_2_O-0.5Er_2_O_3_. The addition of ZnO was found to increase the intensity of the emission at 1.5 µm, as the depolymerized glass network probably improved the regularity of the Er^3+^ site. The glasses underwent heat treatment near their respective glass transition temperature to promote silver clustering. A broad absorption band was detected in the absorption spectrum of the heat-treated glasses, confirming the precipitation of silver-related species. The formation of silver particles can be enhanced by adding ZnO in the glass. The Ag aggregates in the vicinity of Er^3+^ ions were able the increase in the intensity of the emission at 1.5 µm. However, the temperature of the heat treatment should be optimized, as a decrease in the intensity of the emission was observed when the temperature of the heat treatment was slightly increased. Finally, the glasses were heat treated at higher temperatures to promote crystallization. Multiple crystals, the composition of which depends on the glass composition, precipitated from the surface, changing Er^3+^ emission features at 1.5 µm. The addition of ZnO was found to increase the thermal stability of the glass and so to decrease the crystallization tendency of the glass.

## Figures and Tables

**Table 1:** Physical properties of the investigated glasses: density (*ρ*), glass transition temperature (*T_g_*), onset and maximum of crystallization peak temperatures (*T_x_* and *T_p_*), Δ*T* = (*T_x_* − *T_g_*), absorption coefficients (α) and cross-sections (*σ_Abs_*), concentration of Er^3+^ ions.

x	ρ ± 0.02 (g/cm^3^)	*T_g_* ± 3 (°C)	*T_x_* ± 3 (°C)	*T_p_* ± 3 (°C)	Δ*T* ± 6 (°C)	*α* 0.98 μm ± 0.03 (cm^−1^)	*α* 1.53 μm ± 0.06 (cm^−1^)	Er^3+^ (10^19^ ions/cm^3^)	*σ_Abs_* 0.98 μm ± 0.6 (10^−21^ cm^2^)	*σ_Abs_* 1.53 μm ± 1 (10^−21^ cm^2^)
0	2.67	276	378	401	102	0.329	0.875	5.15	6.38	17.0
1.25	2.70	273	383	423	110	0.336	0.876	5.21	6.45	16.8
2.5	2.70	275	390	434	115	0.336	0.903	5.21	6.45	17.3
5	2.73	277	388	446	111	0.352	0.932	5.27	6.68	17.7

**Table 2:** Values of *τ_avg_* (ms) calculated numerically using Equation (2) for the experimental luminescence decay law of Er^3+^: ^4^I_13/2_→^4^I_15/2_ optical transition.

x	as-prepared	Heat Treated for 17 h at		Heat Treated at (*T_g_* + 20 °C) for 17 h and then at (*T_p_* − 40 °C) for	
		(*T_g_* + 10 °C)	(*T_g_* + 20 °C)	1 h	3 h
0	0.88	0.98	0.90	1.14	1.57
1.25	0.80	0.80	0.99	0.91	1.49
2.5	0.99	0.95	0.82	1.04	1.79
5	1.58	1.34	1.39	1.15	1.32

**Table 3:** Values of *τ* (ms) and *β*, obtained by fitting of the experimental luminescence decay law of Er^3+^: ^4^I_13/2_→^4^I_15/_2 optical transition with the stretched exponent function (see Equation (3)).

x	as-prepared	Heat Treated for 17 h at		Heat treated at (*T_g_* + 20 °C) for 17 h and then at (*T_p_* − 40 °C) for	
	*τ (ms)/β*	(*T_g_* + 10 °C)	(*T_g_* + 20 °C)	1 h	3 h
		*τ (ms)/β*	*τ (ms)/β*	*τ (ms)/β*	*τ (ms)/β*
0	0.55/0.74	0.69/0.80	0.56/0.76	0.53/0.65	0.80/0.70
1.25	0.5/0.76	0.52/0.76	0.61/0.75	0.46/0.67	0.46/0.54
2.5	0.66/0.77	0.66/0.81	0.57/0.80	0.63/0.74	0.3/0.44
5	1.26/0.84	1.01/0.83	1.06/0.83	0.71/0.76	0.56/0.65

## Supplementary Materials

The following are available online at https://www.mdpi.com/1996-1944/13/3/527/s1, Figure S1: Emission decay curves and the appropriate fit using the stretched exponential function for investigated glasses and glass ceramics; Figure S2: Normalized emission spectra of Er^3+^:^4^I_13/2_→^4^I_15/2_ optical transition from the glasses prior to and after the heat treatment at *T_g_* + 10 °C and 20 °C for 17 h for x = 0 **(a)**, x = 1.25 **(b)**, x = 2.5 **(c)** and x = 5 **(d)** (*λ_exc_* = 976 nm); Figure S3: Normalized emission spectra of the GC obtained by the heat treatment of x = 0 glass at *T_g_* + 20 °C for 17 h followed by a hold at its respective *T_p_* − 40 °C for 3 h, measured from the surface of a bulk piece and crushed into powder sample.
