# Supplementary material for: Impact of ZnO Addition on Er3+ Near-Infrared Emission, the Formation of Ag Nanoparticles, and the Crystallization of Sodium Fluorophosphate Glass
Source: Materials (Basel). 2020 Jan 22;13(3):527. doi: 10.3390/ma13030527 (PMC7040811; doi:10.3390/ma13030527)
Supplement: Supplementary file 1 [file materials-13-00527-s001.pdf]

materials

Article

# Impact of ZnO Addition on $Er^{3+}$ Near-Infrared Emission, the Formation of Ag Nanoparticles, and the Crystallization of Sodium Fluorophosphate Glass

**Luukas Kuusela [1], Alexander Veber [1,*], Nadia G. Boetti [2], and Laeticia Petit [1]**

1 Photonics Laboratory, Tampere University, Korkeakoulunkatu 3, 33720, Tampere, Finland; luukas.kuusela@tuni.fi (L.K.); laeticia.petit@tuni.fi (L.P.)
2 Fondazione LINKS—Leading Innovation & Knowledge for Society, Via P. C. Boggio 61, 10138 Torino, Italy; nadia.boetti@linksfoundation.com
* Correspondence: alexander.veber@tuni.fi

Received: 19 December 2019; Accepted: 19 January 2020; Published: date

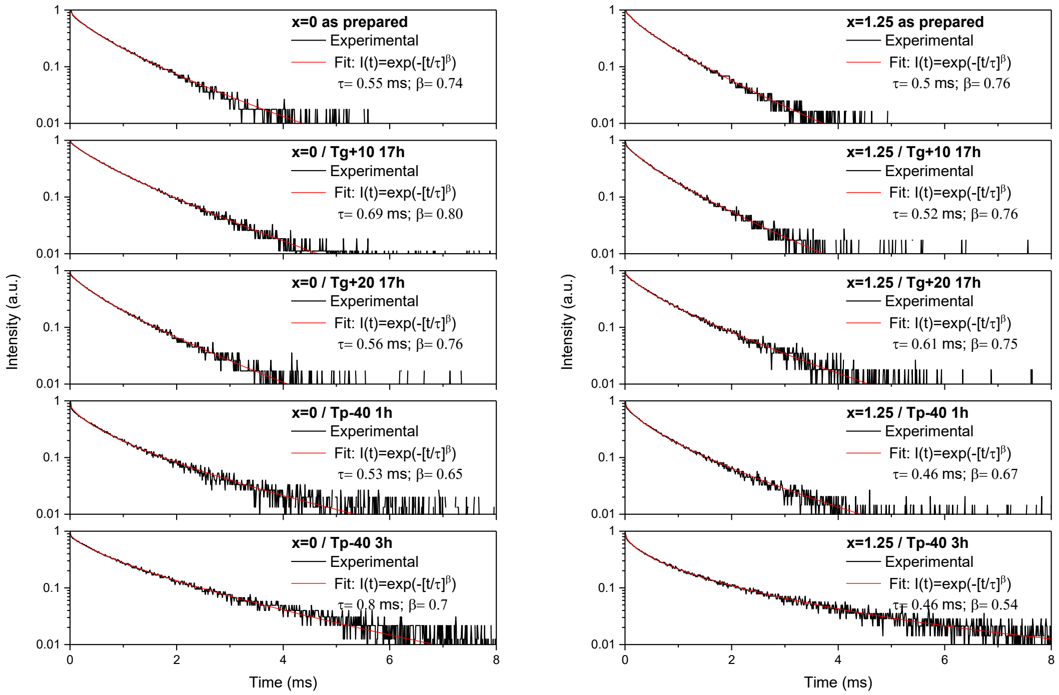

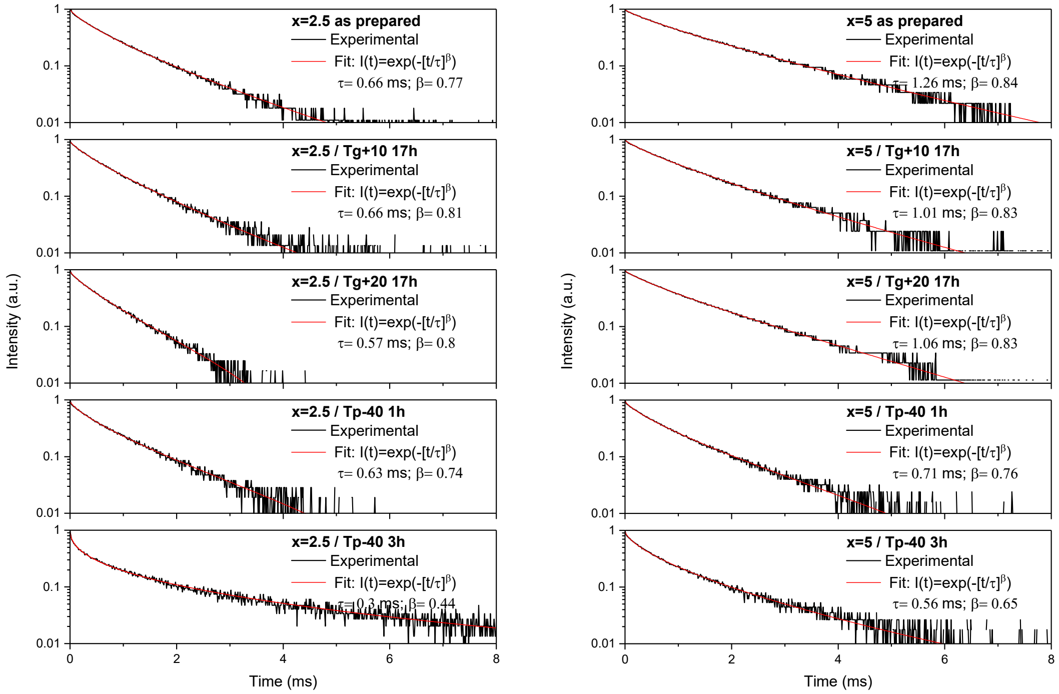


**Figure S1.** Emission decay curves and the appropriate fit using the stretched exponential function for investigated glasses and glass ceramics.

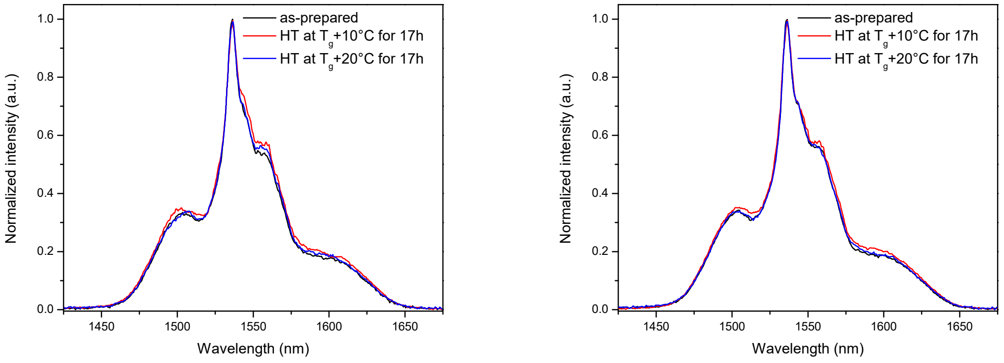


**(a)** **(b)**

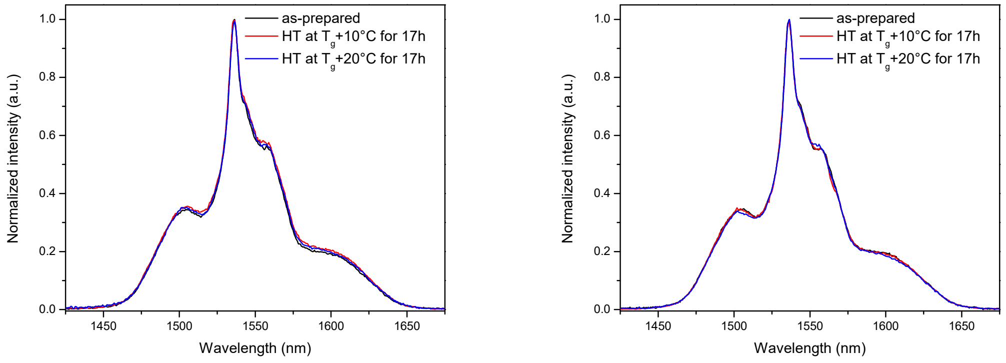


**(c)** **(d)**

**Figure S2.** Normalized emission spectra of $Er^{3+}$: $^4I_{13/2} \rightarrow {}^4I_{15/2}$ optical transition from the glasses prior to and after the heat treatment at $T_g$ + 10°C and 20°C for 17 h for x=0 (a), x=1.25 (b), x=2.5 (c) and x=5 (d) ($\lambda_{exc}$=976 nm). Corresponding integral intensity of the spectra is shown in Figure 6b.

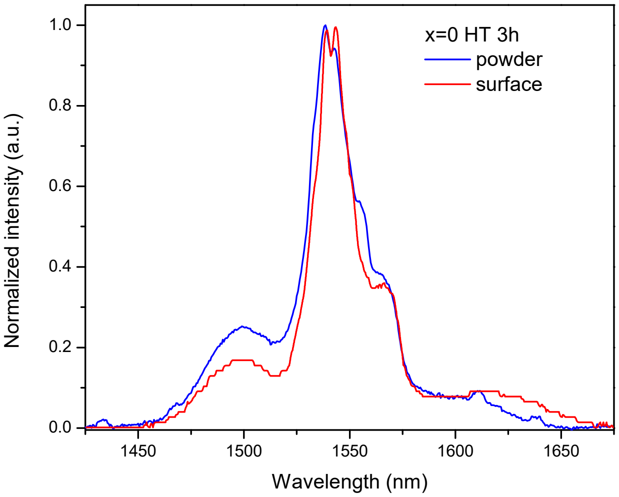


**Figure S3.** Normalized emission spectra of the GC obtained by the heat treatment of x=0 glass at ($T_g$+20°C) for 17 h followed by a hold at its respective ($T_p$-40°C) 3 h measured from the surface of a bulk piece and crushed into powder sample.

© 2020 by the authors. Submitted for possible open access publication under the terms and conditions of the Creative Commons Attribution (CC BY) license (http://creativecommons.org/licenses/by/4.0/).
